# The application value of operating room ventilation with laminar airflow for surgical site infection: A protocol for a systematic review and meta-analysis

**DOI:** 10.1097/MD.0000000000026814

**Published:** 2021-08-13

**Authors:** Yuan-Yuan Liu, Ling-Yun Shi, Yong-Mei Duan, Xiu-Mei Li

**Affiliations:** a Department of Operating Room Disinfection Supply Center, The First Affiliated Hospital of Xinjiang Medical University, Urumqi, Xinjiang, China; b Department of Nursing, The First Affiliated Hospital of Xinjiang Medical University, Urumqi, Xinjiang, China; c Department of No. 2 Coronary Heart Disease, Heart Center, The First Affiliated Hospital of Xinjiang Medical University, Urumqi, Xinjiang, China; d Morphology Center, School of Basic Medicine, Xinjiang Medical University, Urumqi, Xinjiang, China.

**Keywords:** infection, laminar airflow, meta, surgery, ventilation

## Abstract

**Background:**

The presence of biological particles in the air inside operating theatres has the potential to cause severe surgical site infections. Recently, laminar airflow systems have been regarded as a means to reducing surgical site infections using airborne microbes. Still, other publications have argued the benefits of laminar airflow systems, stating the likelihood of adverse effects. Therefore, we will conduct this systematic study to evaluate the applicational value of adopting laminar airflow systems in operating theatres to minimize surgical site infections.

**Methods:**

Reporting of this study adheres to the guidelines of Preferred Reporting Items for Systematic Review and Meta-analysis Protocols. The authors will perform a systematic search on MEDLINE, Web of Science, EMBASE, the China national knowledge infrastructure, and the Cochrane Library from their commencement until June 2021. The search will identify relevant randomized and non-randomized controlled trials that evaluates the applicational value of using laminar airflow ventilation in surgical theatres to minimize surgical site infections. There are no restrictions on language. Two authors will independently screen the identified studies, perform data extraction, and use an appropriate method to evaluate the bias risk in the included studies.

**Results:**

The work done in the present study will enhance the existing literature on the applicational value of laminar airflow ventilation in surgical theatre to reduce surgical site infections.

**Conclusion:**

The outcomes are a reference for healthcare practitioners and patients when making informed decisions regarding care during surgeries.

## 1 Introduction

Globally, surgical site infections are among the most regularly prevalent infections related to health care. These infections lead to a higher incidence of morbidity, longer hospitalization periods, and higher financial expenditures.^[1–4]^ Those who contract surgical site infections face a 60% higher likelihood to be retained in in ICUs. Moreover, these patients face a 5 times the probability of readmission compared to those who do not develop surgical site infections.^[1]^ In combination with longer hospitalization periods, surgical site infections incur higher healthcare costs.^[5–7]^ The implication of airborne pathogens has controversy in the context of its relationship to a higher number of surgical site infections, mainly because the origin of such infections are multifactorial in nature. The ordinary skin flora of inpatients or healthcare practitioners cause over half of all infections after hygienic operations.^[8,9]^

In many countries, ventilation systems are commonly utilized inside operating theatres. In general, 2 air ventilation types are installed in surgical theatres to reduce the airborne pathogens, namely conventional turbulent ventilation and the laminar airflow system.^[10]^ Some healthcare environments recommend terminal high efficiency particulate air filters to be used exclusively in laminar airflow systems.^[11,12]^ Meanwhile, based on technical standards or national regulations, various other countries recommend terminal high efficiency particulate air filters to be used in conventional ventilation systems.^[13]^ Until now, the primary use of laminar airflow is in orthopedic procedures, to reduce surgical infections through airborne pathogens when surgeons insert prosthetic graft materials, such as during artificial joint replacements.^[14,15]^ Most recent studies have argued whether laminar airflow ventilation provides additional benefits, even suggesting that compared to conventional surgical theatres using turbulent ventilation, the incidence of post-surgery surgical site infections could be higher when laminar airflow is used.^[16,17]^ Therefore, this study aims to evaluate the applicational value of surgical theatres using laminar airflow ventilation to minimise surgical site infections.

## 2 Methods

Reporting of this study adheres to the guidelines of Preferred Reporting Items for Systematic Review and Meta-analysis Protocols. The present study is registered under the Open Science Framework (OSF, https://osf.io/).

### 2.1 Criteria for considering studies for review

#### 2.1.1 Types of participants

We shall include all studies involving human participants, regardless of gender, age ethnicity, and healthcare worker groups.

#### 2.1.2 Types of intervention

The authors will include studies describing the applicational value of surgical theatres with laminar airflow ventilation for minimising surgical site infections.

#### 2.1.3 Types of outcome measure

The rate of complication, rate of surgical site infection, and adverse events are the outcomes of this study.

#### 2.1.4 Types of studies

All randomised controlled trials (RCTs) or non-randomised controlled trials, such as case-control, cross-sectional, survey, evaluating the applicational value of surgical theatre ventilation using laminar airflow for surgical site infections will be included.

### 2.2 Search methods for identification of studies

Reporting of this study adheres to the guidelines of Preferred Reporting Items for Systematic Review and Meta-analysis Protocols. The authors will perform a systematic search on MEDLINE, Web of Science, EMBASE, the China national knowledge infrastructure, and the Cochrane Library from their commencement until June 2021. The search will identify relevant randomized and non-randomized controlled trials that evaluates the applicational value of using laminar airflow ventilation in surgical theatres to minimize surgical site infections. The authors will also search World Health Organization International Clinical Trials Registry Platform, Google Scholar, and grey literature to identify all related studies for this review. The literature search uses the following terms: “laminar airflow”, ventilation, and “operating room ventilation”.

### 2.3 Data collection and analysis

#### 2.3.1 Selection of studies

**Figure 1:**
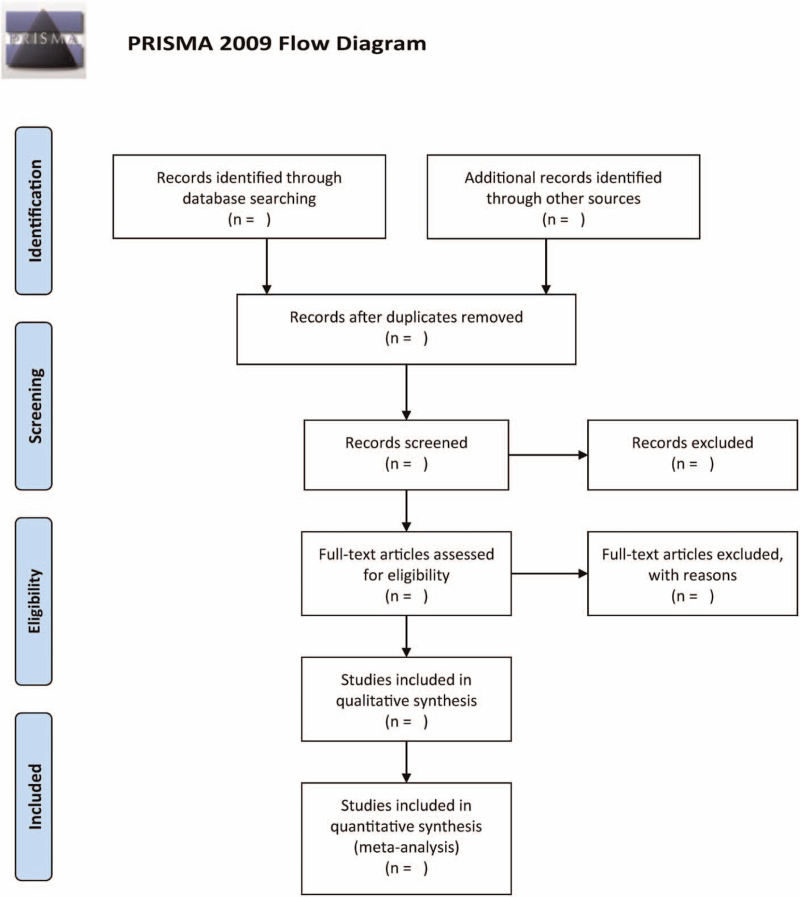
Flowchart of studies selected in the systematic review.

Once duplicate studies are removed, the authors will shortlist eligible research articles. Initially, a pair of authors will independently screen the titles/abstracts to determine suitability. The authors will then proceed to collect the complete-texts of studies that satisfy the eligibility criteria. Afterwards, a pair of independent authors will evaluate the eligibility according to the complete-texts. All disagreements shall be mediated via consultation with another independent author. Figure 1 illustrates the process of selecting eligible studies.

#### 2.3.2 Data extraction and management

A pair of authors will perform manual and independent extraction of data from each study individually and tabulate the extracted data into a predesigned generalized MS Excel worksheet. The following information will be gathered: first author, year of publication, nation, design of study, study period, the total number of operations performed, surgical procedure, evaluation period of surgical site infections, number and types of complications, and outcome evaluation. In the case where data are only available in graphical representation (i.e., plots, figures), we will use Plot Digitizer software to perform data extraction. The authors will perform an extensive study of supplementary materials related to selected studies. Afterwards, the authors will contact respective authors to validate the data extracted and collect any missing/incomplete data.

#### 2.3.3 Assessment of risk of bias in included studies

A pair of authors will autonomously use the Cochrane Risk of Bias Tool to estimate the bias risk.^[18]^ The Newcastle-Ottawa Scale will be used to evaluate the methodological robustness of the included observational articles.^[19]^

#### 2.3.4 Measures of treatment effect

The authors will use the odds ratio and 95% confidence intervals (CIs) for the analysis of dichotomous data. In the event of continuous data outcomes, we will use a weighted mean difference or standard mean difference with 95% for analysis.

#### 2.3.5 Assessment of heterogeneity

We will use the *Q* statistic with the corresponding *P* value and *I*^2^ statistic test to assess the heterogeneity of the included studies. The *I*^2^ statistic will help quantify the portion of total variation in the effect estimation as different outcomes. It is assumed that an *I*^2^ value of 0% reflects no observable heterogeneity, 25% reflects small heterogeneity, 50% reflects average heterogeneity, and 75% reflects high heterogeneity. It is assumed that the included studies are heterogeneous, accounting for clinical heterogeneity. Therefore, a random effects model will be adopted.

#### 2.3.6 Assessment of reporting bias

In the case where the meta-analysis includes a minimum of ten studies, the authors will examine a funnel plot for asymmetry to evaluate the publication bias. Moreover, the authors will extensively evaluate the publication bias adhering to the Egger regression asymmetry test.

#### 2.3.7 Sensitivity analysis

The authors will sequentially remove individual studies from the assessment to conduct a sensitivity analysis to check the robustness of the results.

## 3 Discussion

The present meta-analysis will be the first to synthesize related literature to the applicational value of surgical theatre ventilation with laminar airflow for surgical site infections. It will provide additional information about the applicational value of surgical theatre ventilation that uses laminar airflow to minimise surgical site infections. The results will establish a solid basis for future studies investigating the said area of study through data synthesis. This meta-analysis comprehensively summarizes the rationale and methodologies to provide a complete idea. The use of a comprehensive search strategy is a strength of this protocol. Qualitative and quantitative methods will be used to evaluate the complete data in each analysis. The sources of heterogeneity and different subgroups of the articles will be analysed to completely assess the applicational value of fitting surgical theatres with laminar airflow ventilation to minimize surgical site infections and enhance the credibility of the outcomes. It is hoped that the present meta-analysis will help surgeons, patients, policymakers, and healthcare administrators.

## Author contributions

**Conceptualization:** Yuan-Yuan Liu, Xiu-Mei Li.

**Data curation:** Yuan-Yuan Liu, Ling-Yun Shi, Yong-Mei Duan.

**Formal analysis:** Yuan-Yuan Liu, Ling-Yun Shi, Yong-Mei Duan.

**Funding acquisition:** Yong-Mei Duan, Xiu-Mei Li.

**Investigation:** Yuan-Yuan Liu.

**Methodology:** Yuan-Yuan Liu, Ling-Yun Shi.

**Project administration:** Yuan-Yuan Liu, Yong-Mei Duan, Xiu-Mei Li.

**Resources:** Ling-Yun Shi, Yong-Mei Duan.

**Software:** Yuan-Yuan Liu, Xiu-Mei Li.

**Supervision:** Yong-Mei Duan.

**Validation:** Yuan-Yuan Liu, Yong-Mei Duan, Xiu-Mei Li.

**Visualization:** Ling-Yun Shi, Yong-Mei Duan.

**Writing – original draft:** Yuan-Yuan Liu, Xiu-Mei Li.

**Writing – review & editing:** Xiu-Mei Li.
